# Use of the SNOWED Dataset for Sentinel-2 Remote Sensing of Water Bodies: The Case of the Po River

**DOI:** 10.3390/s24175827

**Published:** 2024-09-08

**Authors:** Marco Scarpetta, Maurizio Spadavecchia, Paolo Affuso, Vito Ivano D’Alessandro, Nicola Giaquinto

**Affiliations:** Department of Electrical and Information Engineering, Polytechnic University of Bari, Via E. Orabona 4, 70125 Bari, Italy; marco.scarpetta@poliba.it (M.S.); v.dalessandro4@phd.poliba.it (V.I.D.); nicola.giaquinto@poliba.it (N.G.)

**Keywords:** remote sensing, satellite monitoring, water bodies segmentation, Sentinel-2 monitoring of environment, river monitoring, water depth sensors, deep learning-based measurements

## Abstract

The paper demonstrates the effectiveness of the SNOWED dataset, specifically designed for identifying water bodies in Sentinel-2 images, in developing a remote sensing system based on deep neural networks. For this purpose, a system is implemented for monitoring the Po River, Italy’s most important watercourse. By leveraging the SNOWED dataset, a simple U-Net neural model is trained to segment satellite images and distinguish, in general, water and land regions. After verifying its performance in segmenting the SNOWED validation set, the trained neural network is employed to measure the area of water regions along the Po River, a task that involves segmenting a large number of images that are quite different from those in SNOWED. It is clearly shown that SNOWED-based water area measurements describe the river status, in terms of flood or drought periods, with a surprisingly good accordance with water level measurements provided by 23 in situ gauge stations (official measurements managed by the Interregional Agency for the Po). Consequently, the sensing system is used to take measurements at 100 “virtual” gauge stations along the Po River, over the 10-year period (2015–2024) covered by the Sentinel-2 satellites of the Copernicus Programme. In this way, an overall space-time monitoring of the Po River is obtained, with a spatial resolution unattainable, in a cost-effective way, by local physical sensors. Altogether, the obtained results demonstrate not only the usefulness of the SNOWED dataset for deep learning-based satellite sensing, but also the ability of such sensing systems to effectively complement traditional in situ sensing stations, providing precious tools for environmental monitoring, especially of locations difficult to reach, and permitting the reconstruction of historical data related to floods and draughts. Although physical monitoring stations are designed for rapid monitoring and prevention of flood or other disasters, the developed tool for remote sensing of water bodies could help decision makers to define long-term policies to reduce specific risks in areas not covered by physical monitoring or to define medium- to long-term strategies such as dam construction or infrastructure design.

## 1. Introduction

In the evolving landscape of climate change, monitoring the extent of water bodies over time has become increasingly crucial for the scientific community due to its importance in various environmental contexts [1,2,3]. Tracking rivers’ flood and drought periods is essential for managing water resources and mitigating natural disasters, while monitoring coastal erosion is vital for protecting coastal infrastructure and ecosystems. Additionally, assessing the health of wetlands, managing agricultural water usage, and tracking glacier retreat, which directly influences sea level rise and freshwater availability, are all critical applications.

Traditional approaches for monitoring water bodies typically involve manual field surveys and data collection from hydrological monitoring stations [4,5]. Although these methods provide high accuracy, they are often costly, time-consuming, and challenging to implement in remote areas, making them impractical for large-scale monitoring. In contrast, remote sensing offers significant advantages, such as global coverage and frequent revisit times [1]. Consequently, the focus of water body monitoring has shifted to satellite sensing data, particularly optical remote sensing, which has seen improvements in spatial resolution and spectral coverage over the past few decades [6]. Various algorithms have been developed to extract surface water extent from satellite imagery, including traditional threshold-based methods [7,8] and more recently proposed machine learning techniques [9,10].

In this context, remote sensing approaches enhanced by deep learning are increasingly being developed to monitor water bodies, thanks to the vast availability of publicly accessible data from programs such as Copernicus [11] and Landsat [12]. Methods proposed in the literature employ deep neural networks (DNNs) for the semantic segmentation of satellite imagery, aiming to identify surface water regions and delineate water bodies [13,14,15,16]. The recent publication of annotated water/land segmentation datasets, such as SWED [17] and SNOWED [18], further exemplifies the progress in water body monitoring methods based on deep learning. These datasets are indispensable for training deep learning segmentation models, enabling more accurate and efficient monitoring of water bodies.

This work presents a novel measurement method for monitoring water bodies using the SNOWED dataset, with a comprehensive application to Italy’s most significant watercourse, the Po River. The primary focus of the study is the development and implementation of a method for spatio-temporal monitoring of water bodies through satellite remote sensing. Unlike traditional approaches that measure metrics such as water discharge or flow velocity, this method focuses on the accurate measurement of water surface area, which is closely linked to water depth allowing for a detailed analysis of changes in water extent over time and space, thus providing valuable insights into the evolution of water distribution, long-term trends, and seasonal variations within various water bodies.

The measurement technique is particularly advantageous for monitoring water bodies that are difficult to assess using in situ methods, making it highly applicable not only to rivers but also to lakes, reservoirs, and smaller water bodies. The application of this method to the Po River, which is already well-monitored through in situ gauging stations, has demonstrated its accuracy, robustness, and potential for broader applications. Overall, the results of this study underscore the versatility of the proposed method and its significant potential impact on water resource management and environmental monitoring, offering a reliable tool for managing and protecting vital water resources in diverse environments.

The paper, which develops early ideas introduced by Scarpetta et al. in 2023 [19], is organized as follows. Section 2 is devoted to material and methods, and describes the employed data sources, the DNN, the remote sensing method, and the validation procedures. Section 3 presents the results, including the metrological assessment of DNN operations, the validation of the monitoring results for the Po River against actual in situ measurements, and a complete space-time monitoring of the Po River across 100 virtual sensing stations. Section 4 presents the conclusions.

## 2. Materials and Methods

### 2.1. Data

In this Section, we describe all data sources used for the remote sensing and for its validation presenting preliminary operations necessary to the satellite image processing, as described in Section 2.2 and Section 2.3.

#### 2.1.1. The SNOWED Dataset

SNOWED, acronym for “Sentinel-2 NOAA Water Edge Dataset”, consist of Sentinel-2 satellite imagery annotated using water edge measurements provided by the National Oceanic and Atmospheric Administration (NOAA) designed for training neural networks for water/land segmentation tasks [18]. Unlike other publicly available datasets [20,21,22,23,24,25,26], the water edges in SNOWED are derived from actual in situ measurements rather than by human analysis of satellite images.

**Figure 1:**
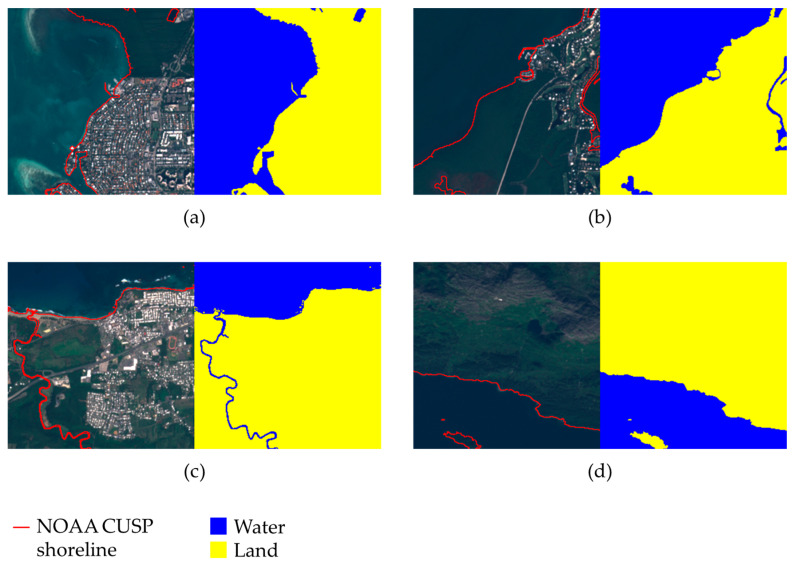
Examples of annotated satellite images from SNOWED. Each subfigure (**a**–**d**) shows the true-color image on the left and the corresponding annotation on the right. NOAA CUSP water edge measurements used to create the annotations are also shown.

SNOWED consists of 4334 samples, each provided as a 256 × 256 sub-tile containing all 13 spectral bands captured by the Sentinel-2 MultiSpectral Instrument (MSI), resampled at a uniform spatial resolution of 10 m. Each sub-tile is accompanied by a water/land segmentation mask, as illustrated in Figure 1, which features four examples highlighting the accuracy and detail of the SNOWED labeling. The examples also show that the dataset primarily focuses on coastal areas, with rivers appearing only occasionally, as seen in Figure 1c. Consequently, using SNOWED for river monitoring represents a highly challenging benchmark for evaluating the dataset’s effectiveness in training general water/land segmentation neural network models.

Like SWED [17] and other datasets examined by Andria et al. [18], SNOWED was created primarily to train neural networks for identifying water bodies in Sentinel-2 images however, it can also be used for other tasks related to water identification in satellite imagery, such as algorithms validation. SNOWED is designed for potential future integration with the SWED dataset (which also consists of 256 × 256 Sentinel-2 images) and possibly with other satellite images datasets for water/land segmentation.

Annotated using in situ measurements available for a limited number of locations, SNOWED is similar to SWED and other datasets (e.g., [24,25,26]), i.e., it contains a few thousand samples, with labels meticulously crafted by actual human effort to ensure high quality. A different kind of datasets (e.g., [20,21,22,23]), consists in collections of a very large number of samples, of the order of hundreds of thousands of images, built by an automatic algorithm which does not use human evaluations, but indexes such the Normalized Difference Water Index (NDWI) [7]. This kind of datasets compensate the lower accuracy of the samples, due to the lack of human intervention, with the larger size, and are usually per se valuable to give environmental evaluations on a global scale.

In the literature, both kind of datasets are used for water bodies measurements and monitoring. For example, Nyberg et al. use a training dataset of 1090 images (512 × 512 pixel) [27]. In contrast, Carbonneau and Bizzi begin training with a large dataset of 740,000 images (224 × 224 pixels) and then refine the model using manually annotated images from 293 location, 15 × 15 km each [28]. Determining the best choice for neural training for a particular sensing problem is certainly an interesting and important topic, but it is clearly beyond the scope of the present work.

#### 2.1.2. EU-Hydro River Network Database

EU-Hydro River Network Database [29] is a dataset providing a photo-interpreted river network for all European countries. The production of EU-Hydro and the derived layers was coordinated by the European Environment Agency (EEA) in the framework of the EU Copernicus program. The river network contained in the dataset is composed of point, line, and polygon objects representing natural rivers and bodies of water, as well as artificial waterways and canals [30].

**Figure 2:**
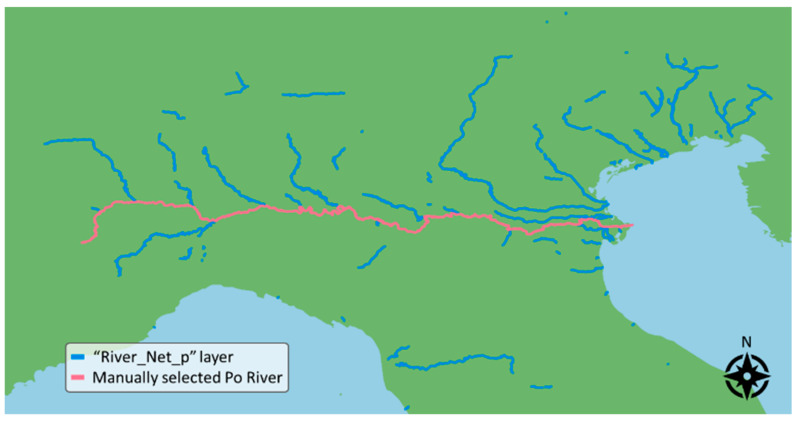
EU-Hydro River Network data relative to North Italy. The Po River basin is highlighted.

The EU-Hydro dataset is divided into packages containing data relative to a single basin. The present work uses the package relative to the Po River, *EU-Hydro-Po-FGDB v013*, and, in particular, the layer *River_Net_p*. This layer provides natural watercourses wider than 50 m in the form of polygons. Figure 2 shows the EU-Hydro mapping of the river network in North Italy, highlighting the Po River basin.

#### 2.1.3. AIPo Water Level Measurements

**Figure 3:**
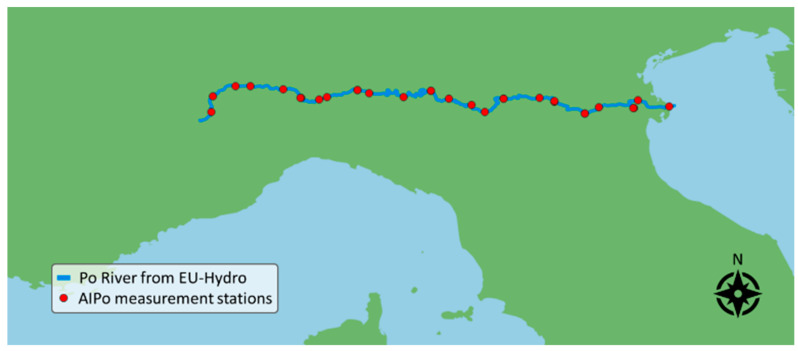
Map of the AIPo gauge stations along the Po River.

The Interregional Agency for the Po River (AIPo) [31] provides water level measurements acquired at 30 gauging stations distributed along the entire path of the river, with seven pairs of them installed in close proximity to each other (few tenths of meters apart). Therefore, on a geographical scale, there are 23 monitored locations, shown in Figure 3. Water level is measured with a frequency of 5–30 min, depending on the specific gauge station.

#### 2.1.4. Sentinel-2 Imagery

The Sentinel-2 mission publicly provides multi-spectral images of the whole world’s land and seawater within 20 km from coasts (except for the Mediterranean Sea which is provided entirely). Satellite images, in 13 spectral bands, are acquired with a revisit time of five days, starting from June 2015 with a spatial resolution ranging from 10 m to 60 m depending on the spectral band. The Sentinel-2 mission is specifically designed for Earth monitoring, and has been selected as the source of satellite imagery due to its technical characteristics and public availability.

Raw swath images are processed according to the Level 1C pipeline, and are provided as 110 × 110 km tiles in UTM projection, each tile having an overlapping region with the neighboring ones. Level 1C has been chosen in order to process easily images in the whole Sentinel-2 history. It is also possible to use the Level 2A pipeline, which contains atmospheric corrections, but this level is not directly available for older Sentinel-2 images.

**Figure 4:**
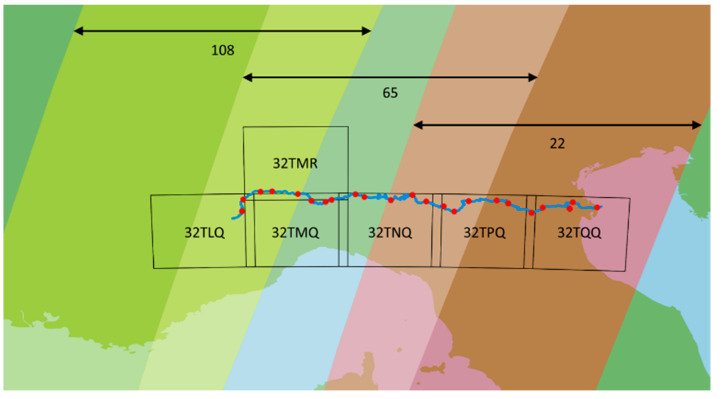
Sentinel 2 orbits covering the Po River surface (108, 65, 22) and tiles selected for analysis.

For the purpose of Po River monitoring, six Sentinel-2 tiles are sufficient to cover the entire basin, as depicted in Figure 4 which also shows that while a tile can be obtained from different orbits, each tile is entirely contained within a single orbit. Using this particular orbit to retrieve the tile is obviously convenient (no need to combine incomplete images of the tile from different orbits). The orbit numbers used for the six tiles are reported in Table 1.

Tiles are filtered and retrieved by using the Copernicus Data Space Ecosystem [32], which allows selection based on tile identifier, orbit number, and cloud cover (as well as other parameters that we do not use). We select tiles with identifier and orbit number in Table 1, and with cloud cover of less than 40%. A greater limit for cloud cover (e.g., 60%) can also be used, with the drawback of increasing the quantity of downloaded data and processing time.

In the operations detailed in the subsequent sections, tiles are not analyzed as whole entities; instead, sub-tiles of 256 × 256 pixel are considered. Additionally, a sub-tile is analyzed only if the corresponding Sentinel 2A scene classification contains less than 5% of clouds or defective pixels.

### 2.2. Neural Network for Water/Land Segmentation of Satellite Images

The DNN performing automatic segmentation of Sentinel-2 imagery into water and non-water areas uses the well-known and widely recognized U-Net architecture [33,34] which comprises a contracting and expanding path, designed to facilitate robust feature extraction and accurate localization of these areas.

The input of the neural network is a 256 × 256 image with 13 channels, corresponding to the Sentinel-2 Level 1C bands, while the output is a 256 × 256 × 2 matrix, with the values of probability of water and non-water, respectively, in each of the two channels. Each pixel is classified as water when the associated probability is greater than 50%.

**Figure 5:**
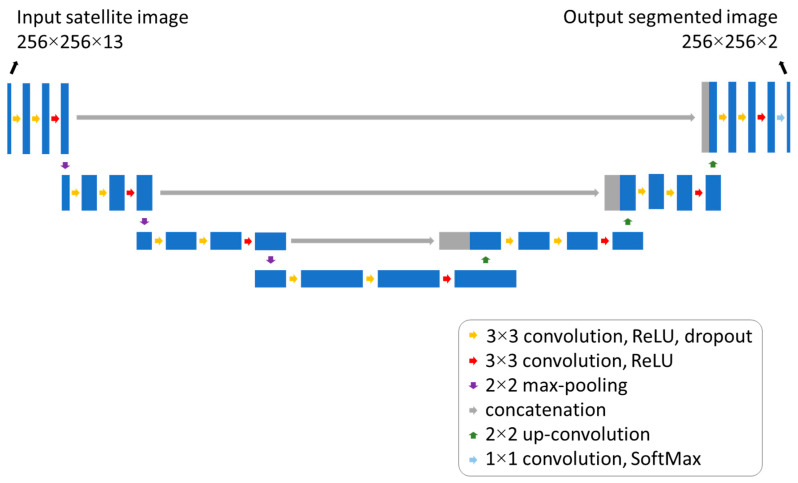
Neural network architecture.

The U-Net architecture is visually represented in Figure 5, offering an insight into the network’s structural design and connectivity. It consists of a contracting path for capturing context, and a symmetric expanding path for precise segmentation. The contracting path has three consecutive convolutional blocks with 32 filters, each of 3 × 3 kernel size, ReLU activation, and “He” normal initialization, followed by dropout layers with a rate of 20% for regularization. A max-pooling layer is used to reduce spatial dimensions. This structure is replicated three times, doubling twice the number of filters (from 32 to 128). These blocks are followed by a bottleneck block, composed of three convolutional layers with 256 filters, kernel size of 3 × 3 and dropout layers with a rate of 20%. The expanding path of the network is specular with respect to the contracting path, but the convolutional layers are substituted by transposed convolutional ones. The contracting path and the expanding path are connected using skip-connection layers. Lastly, the output layer of the network is a convolutional layer with a SoftMax activation function and two filters to obtain a binary segmentation of the input image.

The Adam optimizer has been used to train the neural network [35], employing the binary cross-entropy loss function, appropriate for binary segmentation tasks [36]. To augment the generalization capabilities of the model, data augmentation techniques, including rotation and flipping, have been applied during training. The model is trained on 90% of samples of SNOWED for 300 epochs, with a batch size of 32. The remaining 10% of samples are used for validation, whose results are reported in Section 3.1.

### 2.3. Sensing Algorithm

**Figure 6:**
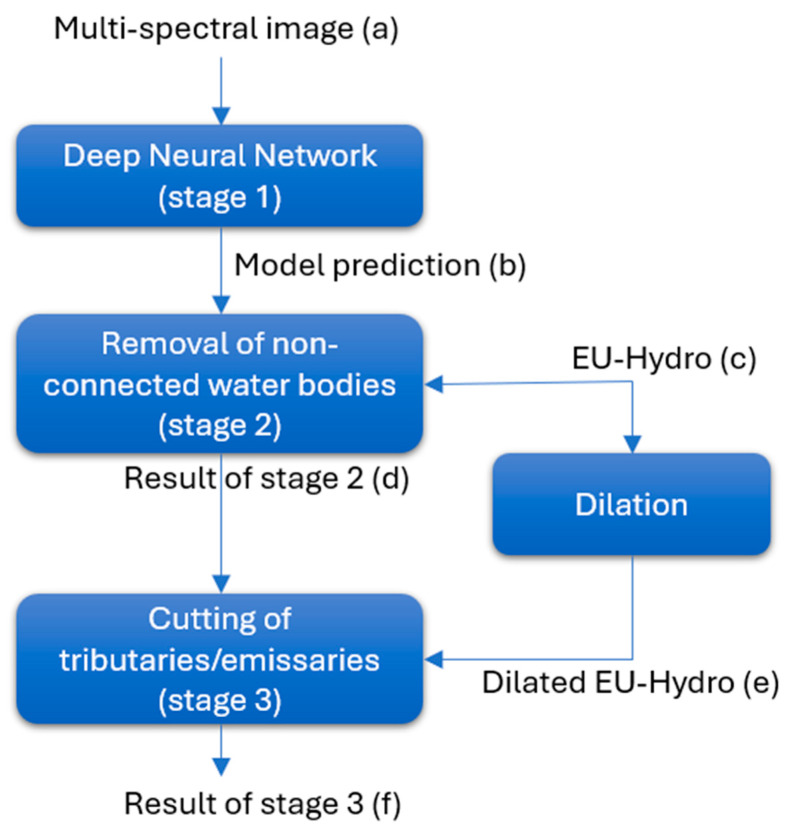
Sensing algorithm flowchart.

**Figure 7:**
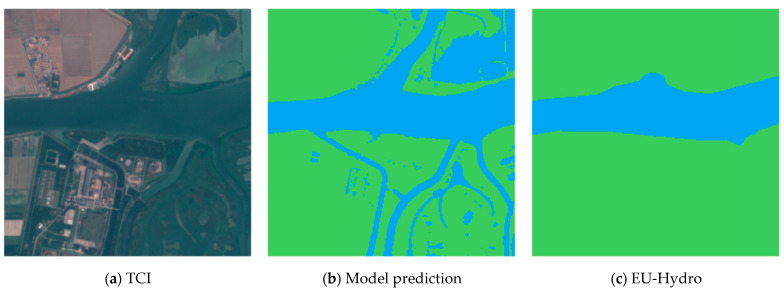

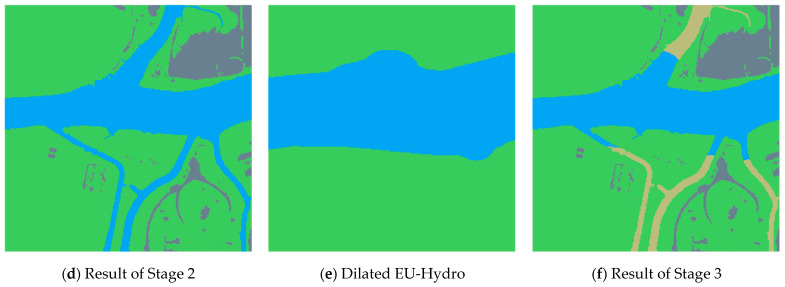
Images involved in the sensing algorithm of Figure 6.

The sensing algorithm is described in Figure 6, which depicts the sequence of operations, and in Figure 7, showing actual images involved in the processing of a specific sub-tile.

The algorithm is a three-stage process, applied to the 13-layer multispectral Sentinel-2 image (256 × 256 pixel sub-tile), represented by a single TCI image in Figure 7a.

In the first stage, the most important, the sub-tile is processed by the DNN, and the water in the image is identified. The result of this stage is represented in Figure 7b. As depicted, the DNN prediction also includes water bodies that are not part of the river basin. The second and the third stage have the purpose of excluding these water areas, which are not related to the river regime, by using the “nominal” river area provided by EU-Hydro, shown in Figure 7c.

In the second stage, all non-connected water regions in the DNN prediction are analyzed. The regions that do not intersect the EU-Hydro river area are considered disconnected from the river basin and are discarded (Figure 7d), leaving in the map only the river with its tributaries/emissaries.

In the third stage, tributaries and emissaries are removed by intersecting the output from the second stage with the EU-Hydro area, which has been previously subjected to morphological dilation using a 40 × 40 kernel (Figure 7e). This dilation operation expands the water area in the EU-Hydro binary mask by setting to water any pixel that has at least one water pixel within its 40 × 40 neighborhood. Using the dilated EU-Hydro area for the intersection ensures better coverage of the main river course while retaining only the initial segments of tributaries and emissaries (Figure 7f), which exhibit the same water regime—whether drought or fullness—as the main river.

### 2.4. Methodology for Assessing the Performance of the DNN

The first and most important stage of the algorithm in Figure 6 is the identification of water regions performed by the DNN. Assessing its effectiveness is particularly important, since this part of the algorithm can be used for segmenting water and land areas across diverse geographical contexts. The performance of the DNN has been evaluated using different metrics computed using the samples of the SNOWED dataset selected as validation set, i.e., 443 images (10% of the whole dataset).

**Figure 8:**
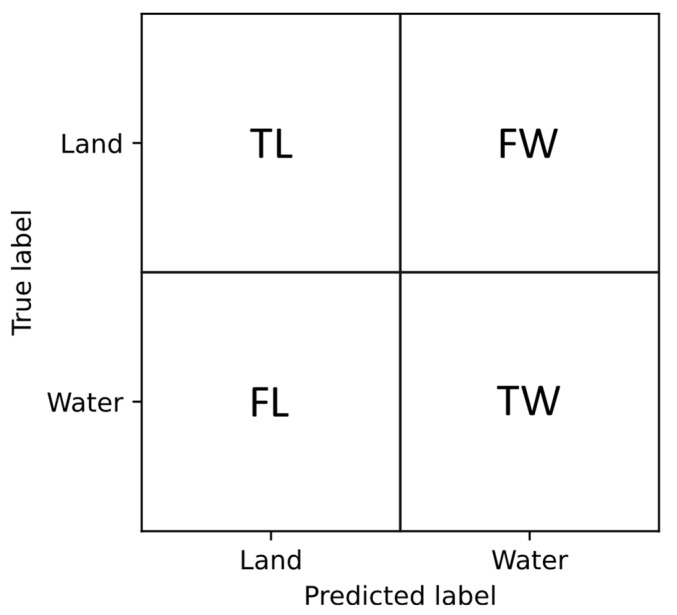
Confusion matrix for water/land segmentation problems.

The first set of metrics is derived from the confusion matrix, which compares the predicted classes with the true classes for each pixel. For clarity, the structure of the confusion matrix is shown in Figure 8. The diagonal elements of the matrix represent correctly classified pixels—true land (*TL*) and true water (*TW*). In contrast, the off-diagonal elements represent misclassified pixels: false water (*FW*) and false land (*FL*).

The following metrics are computed using the elements of the confusion matrix.

- Accuracy, which measures the proportion of correctly classified pixels out of the total number of pixels:

$$ACC=\frac{TL+TW}{TL+TW+FL+FW}$$

- Precision, also known as Positive Predictive Value (*PPV*). For the water class, it measures the proportion of pixels predicted as water that are correctly classified:

$${PPV}_{W}=\frac{TW}{TW+FW}$$

- Recall, also known as True Positive Rate (*TPR*). For the water class, it measures the proportion of actual water pixels that are correctly identified.

$${TPR}_{W}=\frac{TW}{TW+FL}$$

- *F*1 score which is the harmonic mean of precision and recall, providing a balanced measure that considers both false positives and false negatives:

$${F1}_{W}=⁠\frac{2\;PPV_{W}\;TPR_{W}}{PPV_{W}+TPR_{W}}$$

- Intersection over Union (*IoU*), which is widely used in semantic segmentation tasks [37,38,39,40]. It measures the overlap between the predicted and the true mask for each class. The *IoU* for the water class is calculated as:

$$IoU_{W}=\frac{TW}{TW+FW+FL}$$

Precision, Recall, *F*1 score, and *IoU* are also computed for the land class, and then the mean values of these metrics across the two classes are taken.

The last metric is based on the water area measurement error (*WAME*), defined according to the GUM convention [41]:

WAME=measuredwaterarea−referencewaterarea

where the reference water area is provided by the labels of the images in the validation set. WAMEs assess the DNN as an actual sensor for measuring the amount of water in the satellite image. Besides, water area errors help understanding the applicability of the algorithm for measurements different from river monitoring, and the potential for accuracy improvements.

The accuracy of the DNN water area measurements is evaluated by computing a symmetric interval that encompasses 90% of all WAMEs of the validation set. The result is given both in pixels and in surface measurement units (squared kilometers).

### 2.5. Comparison between Remote Water Area Measurements and Local Water Depth Measurements

In addition, to evaluate the DNN water/land segmentation performance as described in Section 2.4, the complete sensing algorithm presented in Section 2.3 is validated using data from the Po River. This validation involves comparing water area measurements from the SNOWED-based system with depth variations provided by AIPo [31]. Specifically, depth variations recorded by AIPo at locations shown in Figure 3 are compared with water area changes measured by the SNOWED-based system at the same locations. Although water area and water depth are distinct quantities, both are hydraulic variables that determine and are influenced by local water volume. As a matter of fact, both water surface [42,43] and water depth [44] have been used to monitor spatiotemporal variations in river volumes and flows. This comparison thus offers a challenging and reliable method to assess the effectiveness of the remote sensing approach for meaningful and accurate river monitoring.

### 2.6. Virtual Gauge Stations along the Po River

**Figure 9:**
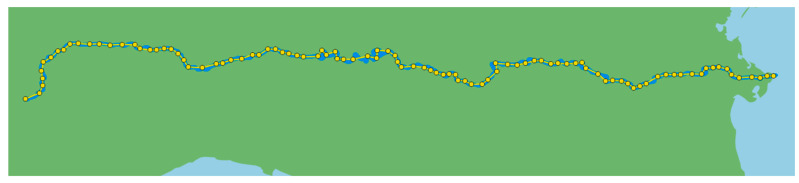
Map of the virtual gauge stations along the Po River.

A set of 100 virtual gauge stations, corresponding to an equal number of Sentinel-2 sub-tiles, is implemented to achieve a comprehensive monitoring of the entire Po River. As shown in Figure 9, these stations are positioned randomly within the EU-Hydro polygon of the river using QGIS 3.28 software, with a minimum separation of 0.04 degrees between each pair of points.

The river monitoring has been performed by downloading the Sentinel-2 images specified in Section 2.1.3, and then extracting 256 × 256 sub-tiles centered on the virtual gauge stations. Then, the surface water area has been measured for all the sub-tiles, using the sensing algorithm described in Section 2.3.

## 3. Results and Discussion

### 3.1. Assessment of Remote Sensing System Using the SNOWED Validation Set

**Figure 10:**
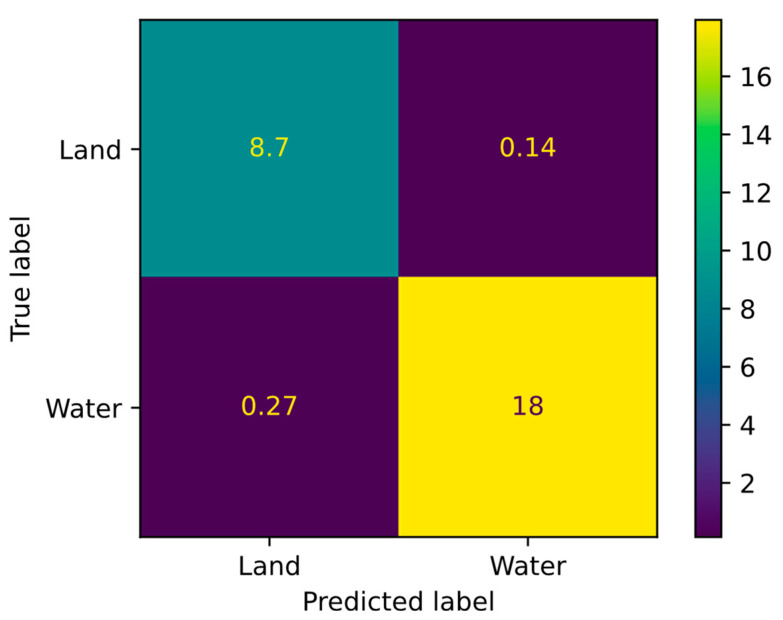
Confusion matrix for the water/land segmentation on the SNOWED validation set (values in megapixels).

The performance of the DNN has been evaluated as described in Section 2.4 by calculating the confusion matrix across the 433 images selected as validation set from the SNOWED dataset (Figure 10), with the corresponding metrics presented in Table 2. The results show that all metrics are close to their maximum values. Additionally, the metrics for the water class, which is the class of primary interest, are higher than those for the land class with the DNN achieving a mean *IoU* of 96.7%, indicating a high degree of overlap between the predicted and true masks.

As regards errors in measuring water areas, the symmetric interval encompassing 90% of all evaluated WAMEs has been found to be ±1759 pixels, equivalent to ±0.18 km^2^. These figures provide a synthetic yet informative assessment of the DNN accuracy in making measurements.

### 3.2. Assessment of the Final Measurements by Comparison with Measurements by Local Depth Sensors

**Figure 11:**
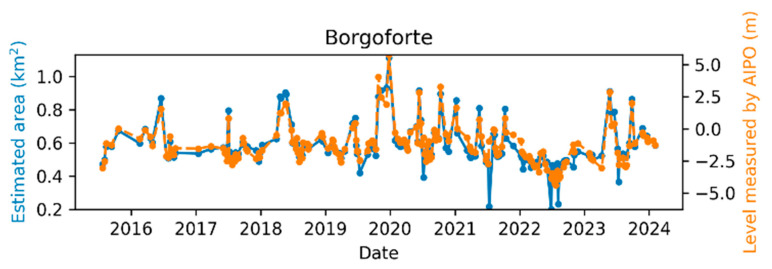
Po River monitoring in Borgoforte.

**Figure 12:**
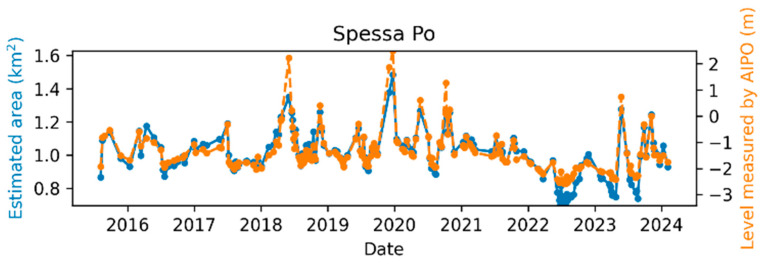
Po River monitoring in Spessa Po.

**Figure 13:**
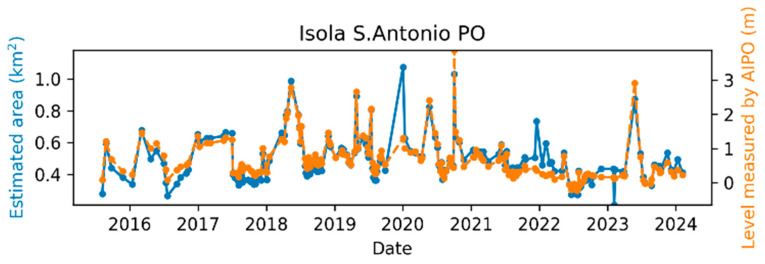
Po River monitoring in Isola S. Antonio Po.

**Figure 14:**
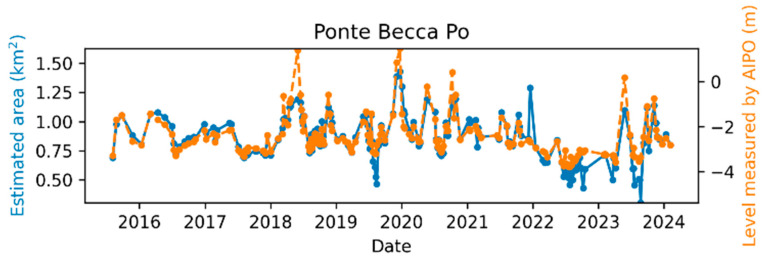
Po River monitoring in Ponte Becca PO.

**Figure 15:**
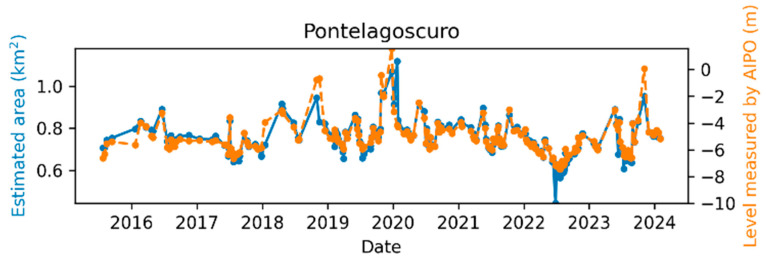
Po River monitoring in Pontelagoscuro.

**Figure 16:**
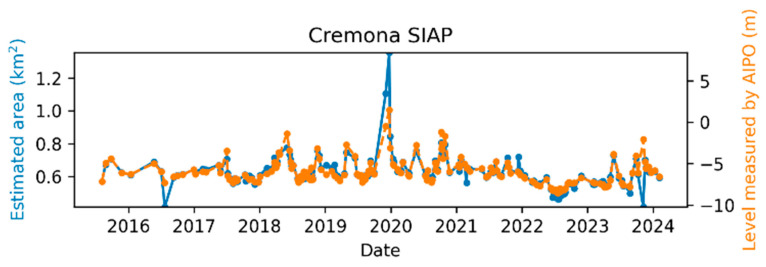
Po River monitoring in Cremona SIAP.

The comparison between the AIPo river depth measurements and those from the remote sensing algorithm is illustrated in Figure 11, Figure 12, Figure 13, Figure 14, Figure 15 and Figure 16, which depict the results obtained at six different AIPo gauge stations, namely, Borgoforte, Spessa Po, Isola Sant’Antonio PO, Ponte Becca PO, Pontelagoscuro, and Cremona SIAP demonstrating a highly consistent correspondence between remote and local monitoring, with occasional outliers that are easily identifiable in Figure 11, Figure 12, Figure 13, Figure 14, Figure 15 and Figure 16.

**Figure 17:**
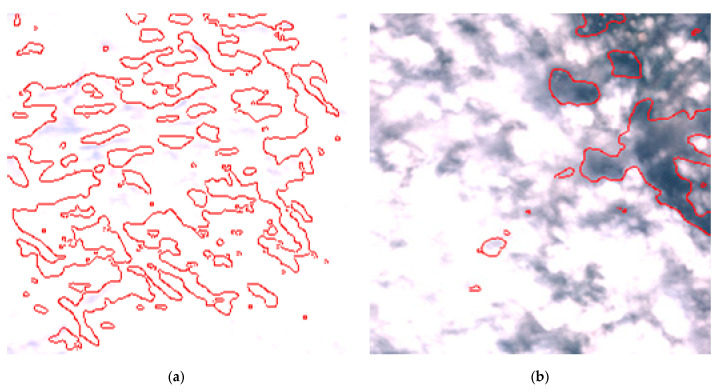
Explanation of outliers in remote monitoring results. The segmentation performed by the DNN with adverse weather conditions are shown in red. (**a**) Ponte Becca Po, 17 December 2021 (cloudy weather); (**b**) Cremona SIAP, 7 November 2023, (cloudy weather).

By reviewing the satellite images on the dates and positions of the outliers, it is evident that these anomalies are due to adverse weather conditions such as clouds, snow, or fog. Despite discarding sub-tiles with excessive cloud cover, as outlined in Section 2.1.3, the Sentinel-2A scene classification algorithm occasionally fails to identify defective sub-tiles. Consequently, sometimes it is impossible for the DNN (or any algorithm having access only to Sentinel-2 imagery) to identify water areas. Figure 17 shows the TCI satellite images for two of such cases. In a more advanced implementation of the method, cloud detection can be easily enhanced using deep learning techniques, which are already well-established in the literature [45,46], to identify and discard all sub-tiles containing clouds.

Apart from the outliers, the remote monitoring results are very satisfactory, demonstrating that the remote sensing method is clearly able to provide information about the river regime comparable to that obtained from local gauge stations.

### 3.3. Space-Time Po River Monitoring

**Figure 18:**
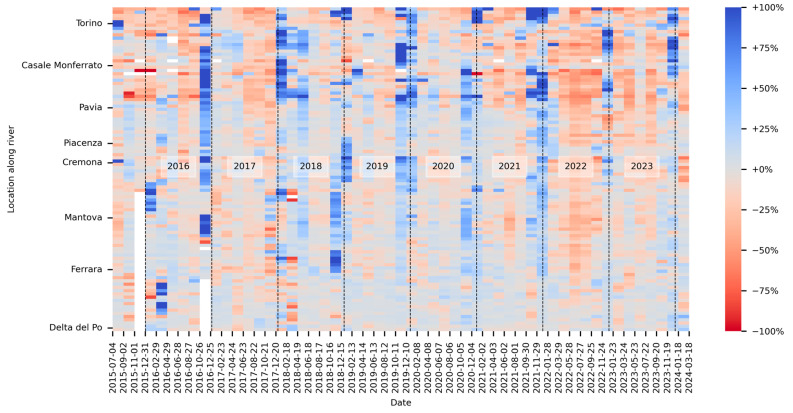
Percentage variation of water area over time, along the Po River. White rectangles denote periods with no available data, indicating the absence of satellite images meeting cloud coverage requirements for those times and locations.

The outcomes of the Po River monitoring by the 100 virtual stations described in Section 2.6, spanning the 9-year period from 2015 to 2024, are shown in the heatmap in Figure 18.

For each virtual gauge station, Figure 18 shows the percentage variation of the measured water area, with respect to the mean water area measured by the same virtual station over the entire 9-year monitoring period. The temporal axis divides the monitoring period into intervals of 60 days each; therefore, each rectangle on the map represents the mean of the measurements taken during the 60-day period defined by the dates on the x-axis. Since the revisit time of the Sentinel-2 mission is five days, each rectangle in the map represents the mean of a maximum of 12 measurements.

Distinct colors within the heatmap in Figure 18 used as indicators of diverse hydrological conditions. Regions colored in red are related to periods of drought, indicating a reduction in water surface levels during those specific intervals. Conversely, regions shaded in blue represent periods of elevated water surface levels, which could suggest potential flood occurrences.

This visual presentation offers a comprehensive insight into the temporal and spatial dynamics of the Po River, facilitating a thorough evaluation of droughts and floods at various sites along the river basin. For example, the Figure 18 shows very clearly the 2022 drought, which has been found to be the worst in the past two centuries [47].

## 4. Conclusions

The paper presents (i) a remote sensing method, based on a neural network for water/land segmentation trained with the SNOWED dataset, for monitoring river regimes using Sentinel-2 imagery, (ii) actual results obtained from the application of the method to Italy’s main watercourse, the Po River, (iii) the validation of the results obtained by the comparison of remote measurements with measurements from local sensors, and (iv) an overall monitoring of the Po River for its whole length and for nine years.

A key outcome of the study is the demonstration of the effectiveness of the SNOWED dataset, which is not specifically constructed to monitor rivers, since the method provides river regime information comparable to that obtained from local sensing stations, whenever weather conditions allow for satellite observations. Additionally, the method’s accuracy in measuring water area has been rigorously assessed, making it suitable for other satellite-based water body measurements. Specifically, the core DNN achieves a mean IoU of 96.7% and a water area measurement error within ±0.18 km^2^ in 90% of cases. The application of the method to the Po River has enabled comprehensive monitoring at 100 virtual sensing stations, covering the period from the inception of the Sentinel-2 mission in 2015 to the present day.

In conclusion, the research clearly demonstrates that the proposed method has the potential, with minor enhancements, for application to comprehensive monitoring of water surfaces of rivers, lakes, and other water bodies on a global scale. This is a key activity for a better understanding and management of water systems, and in general of the evolution of the environmental conditions.

## Figures and Tables

**Table 1:** Selected Tiles and Orbits.

Tile Identifier	Relative Orbit Number
32TLQ	108
32TMR	65
32TMQ	65
32TNQ	65
32TPQ	22
32TQQ	22

**Table 2:** Metrics calculated from the confusion matrix.

	ACC	PPV	TPR	F1	IoU
Land	-	97.0%	98.5%	97.7%	95.6%
Water	-	99.2%	98.5%	98.9%	97.8%
Mean	98.5%	98.1%	98.5%	98.3%	96.7%

## Data Availability

Publicly available Sentinel-2 data products used in this study can be found at: https://dataspace.copernicus.eu/ (accessed on 5 June 2024). Publicly available Po River water level measurements used in this study can be found at: https://www.agenziapo.it/content/monitoraggio-idrografico-0 (accessed on 26 January 2024).

## References

[1] Li J., Ma R., Cao Z., Xue K., Xiong J., Hu M., Feng X. (2022). Satellite Detection of Surface Water Extent: A Review of Methodology. Water.

[2] Khandelwal A., Karpatne A., Marlier M.E., Kim J., Lettenmaier D.P., Kumar V. (2017). An Approach for Global Monitoring of Surface Water Extent Variations in Reservoirs Using MODIS Data. Remote Sens. Environ..

[3] Yang X., Qin Q., Yésou H., Ledauphin T., Koehl M., Grussenmeyer P., Zhu Z. (2020). Monthly Estimation of the Surface Water Extent in France at a 10-m Resolution Using Sentinel-2 Data. Remote Sens. Environ..

[4] Segovia-Cardozo D.A., Rodríguez-Sinobas L., Canales-Ide F., Zubelzu S. (2021). Design and Field Implementation of a Low-Cost, Open-Hardware Platform for Hydrological Monitoring. Water.

[5] Kuang K.S.C., Quek S.T., Maalej M. (2008). Remote Flood Monitoring System Based on Plastic Optical Fibres and Wireless Motes. Sens. Actuators A Phys..

[6] Huang C., Chen Y., Zhang S., Wu J. (2018). Detecting, Extracting, and Monitoring Surface Water From Space Using Optical Sensors: A Review. Rev. Geophys..

[7] McFeeters S.K. (1996). The Use of the Normalized Difference Water Index (NDWI) in the Delineation of Open Water Features. Int. J. Remote Sens..

[8] Xu H. (2006). Modification of Normalised Difference Water Index (NDWI) to Enhance Open Water Features in Remotely Sensed Imagery. Int. J. Remote Sens..

[9] Acharya T.D., Subedi A., Lee D.H. (2019). Evaluation of Machine Learning Algorithms for Surface Water Extraction in a Landsat 8 Scene of Nepal. Sensors.

[10] Hibjur Rahaman M., Roshani, Masroor M., Sajjad H. (2023). Integrating Remote Sensing Derived Indices and Machine Learning Algorithms for Precise Extraction of Small Surface Water Bodies in the Lower *Thoubal* River Watershed, India. J. Clean. Prod..

[11] Homepage|Copernicus. https://www.copernicus.eu/en.

[12] Landsat Science. https://landsat.gsfc.nasa.gov/.

[13] Tambe R.G., Talbar S.N., Chavan S.S. (2021). Deep Multi-Feature Learning Architecture for Water Body Segmentation from Satellite Images. J. Vis. Commun. Image Represent..

[14] Boston T., Van Dijk A., Larraondo P.R., Thackway R. (2022). Comparing CNNs and Random Forests for Landsat Image Segmentation Trained on a Large Proxy Land Cover Dataset. Remote Sens..

[15] Yuan K., Zhuang X., Schaefer G., Feng J., Guan L., Fang H. (2021). Deep-Learning-Based Multispectral Satellite Image Segmentation for Water Body Detection. IEEE J. Sel. Top. Appl. Earth Obs. Remote Sens..

[16] Wieland M., Martinis S., Kiefl R., Gstaiger V. (2023). Semantic Segmentation of Water Bodies in Very High-Resolution Satellite and Aerial Images. Remote Sens. Environ..

[17] Seale C., Redfern T., Chatfield P., Luo C., Dempsey K. (2022). Coastline Detection in Satellite Imagery: A Deep Learning Approach on New Benchmark Data. Remote Sens. Environ..

[18] Andria G., Scarpetta M., Spadavecchia M., Affuso P., Giaquinto N. (2023). SNOWED: Automatically Constructed Dataset of Satellite Imagery for Water Edge Measurements. Sensors.

[19] Scarpetta M., Ragolia M.A., Spadavecchia M., Affuso P., Giaquinto N. The SNOWED Dataset and Its Application to Po River Monitoring Through Satellite Images. Proceedings of the 2023 IEEE International Conference on Metrology for eXtended Reality, Artificial Intelligence and Neural Engineering (MetroXRAINE).

[20] Feng M., Sexton J.O., Channan S., Townshend J.R. (2016). A Global, High-Resolution (30-m) Inland Water Body Dataset for 2000: First Results of a Topographic–Spectral Classification Algorithm. Int. J. Digit. Earth.

[21] Pekel J.-F., Cottam A., Gorelick N., Belward A.S. (2016). High-Resolution Mapping of Global Surface Water and Its Long-Term Changes. Nature.

[22] Isikdogan F., Bovik A.C., Passalacqua P. (2017). Surface Water Mapping by Deep Learning. IEEE J. Sel. Top. Appl. Earth Obs. Remote Sens..

[23] Isikdogan L.F., Bovik A., Passalacqua P. (2020). Seeing Through the Clouds with DeepWaterMap. IEEE Geosci. Remote Sens. Lett..

[24] QueryPlanet Water Segmentation Data Set. http://queryplanet.sentinel-hub.com/index.html?prefix=/#waterdata.

[25] Yang T., Jiang S., Hong Z., Zhang Y., Han Y., Zhou R., Wang J., Yang S., Tong X., Kuc T. (2020). Sea-Land Segmentation Using Deep Learning Techniques for Landsat-8 OLI Imagery. Mar. Geod..

[26] Erdem F., Bayram B., Bakirman T., Bayrak O.C., Akpinar B. (2021). An Ensemble Deep Learning Based Shoreline Segmentation Approach (WaterNet) from Landsat 8 OLI Images. Adv. Space Res..

[27] Nyberg B., Henstra G., Gawthorpe R.L., Ravnås R., Ahokas J. (2023). Global Scale Analysis on the Extent of River Channel Belts. Nat. Commun.

[28] Carbonneau P.E., Bizzi S. (2024). Global Mapping of River Sediment Bars. Earth Surface Processes and Landforms.

[29] EU-Hydro—River Network Database—Copernicus Land Monitoring Service. https://land.copernicus.eu/imagery-in-situ/eu-hydro/eu-hydro-river-network-database.

[30] Gallaun H., Dohr K., Puhm M., Stumpf A., Hugé J. (2021). EU-Hydro—River Net User Guide 1.3.

[31] Monitoraggio Idrografico|AIPO—Agenzia Interregionale per Il Fiume PO. https://www.agenziapo.it/content/monitoraggio-idrografico-0.

[32] Ecosystem C.D.S. Copernicus Data Space Ecosystem|Europe’s Eyes on Earth. https://dataspace.copernicus.eu/.

[33] Ronneberger O., Fischer P., Brox T., Navab N., Hornegger J., Wells W.M., Frangi A.F. (2015). U-Net: Convolutional Networks for Biomedical Image Segmentation. Proceedings of the Medical Image Computing and Computer-Assisted Intervention—MICCAI 2015.

[34] Scarpetta M., Affuso P., De Virgilio M., Spadavecchia M., Andria G., Giaquinto N. Monitoring of Seagrass Meadows Using Satellite Images and U-Net Convolutional Neural Network. Proceedings of the 2022 IEEE International Instrumentation and Measurement Technology Conference (I2MTC).

[35] Kingma D.P., Ba J. (2014). Adam: A Method for Stochastic Optimization. arXiv.

[36] Jadon S. A Survey of Loss Functions for Semantic Segmentation. Proceedings of the 2020 IEEE Conference on Computational Intelligence in Bioinformatics and Computational Biology (CIBCB).

[37] Pedrayes O.D., Lema D.G., García D.F., Usamentiaga R., Alonso Á. (2021). Evaluation of Semantic Segmentation Methods for Land Use with Spectral Imaging Using Sentinel-2 and PNOA Imagery. Remote Sens..

[38] Ayala C., Aranda C., Galar M. (2021). Towards Fine-Grained Road Maps Extraction Using Sentinel-2 Imagery. ISPRS Ann. Photogramm. Remote Sens. Spat. Inf. Sci..

[39] D’Alessandro V.I., Palma L.D., Attivissimo F., Nisio A.D., Lanzolla A.M.L. U-Net Convolutional Neural Network for Multisource Heterogeneous Iris Segmentation. Proceedings of the 2023 IEEE International Symposium on Medical Measurements and Applications (MeMeA).

[40] Kotaridis I., Lazaridou M. (2022). Semantic Segmentation Using a UNET Architecture on Sentinel-2 Data. Int. Arch. Photogramm. Remote Sens. Spat. Inf. Sci..

[41] Joint Committee for Guides in Metrology (2008). Evaluation of Measurement Data—Guide to the Expression of Uncertainty in Measurement (GUM 1995 with Minor Corrections).

[42] Zhou J., Ke L., Ding X., Wang R., Zeng F. (2024). Monitoring Spatial–Temporal Variations in River Width in the Aral Sea Basin with Sentinel-2 Imagery. Remote Sens..

[43] Kazemi Garajeh M., Haji F., Tohidfar M., Sadeqi A., Ahmadi R., Kariminejad N. (2024). Spatiotemporal Monitoring of Climate Change Impacts on Water Resources Using an Integrated Approach of Remote Sensing and Google Earth Engine. Sci. Rep..

[44] Dubey A.K., Gupta P.K., Dutta S., Singh R.P. (2015). An Improved Methodology to Estimate River Stage and Discharge Using Jason-2 Satellite Data. J. Hydrol..

[45] Wright N., Duncan J.M.A., Callow J.N., Thompson S.E., George R.J. (2024). CloudS2Mask: A Novel Deep Learning Approach for Improved Cloud and Cloud Shadow Masking in Sentinel-2 Imagery. Remote Sens. Environ..

[46] Pang S., Sun L., Tian Y., Ma Y., Wei J. (2023). Convolutional Neural Network-Driven Improvements in Global Cloud Detection for Landsat 8 and Transfer Learning on Sentinel-2 Imagery. Remote Sens..

[47] Montanari A., Nguyen H., Rubinetti S., Ceola S., Galelli S., Rubino A., Zanchettin D. (2023). Why the 2022 Po River Drought Is the Worst in the Past Two Centuries. Sci. Adv..

